# Adipose‐Derived Stem Cells From Human Lumbar Subcutaneous Tissue in Obesity: Inflammatory Microenvironment Promotes Differentiation Capacity

**DOI:** 10.1155/sci/2552466

**Published:** 2026-05-07

**Authors:** Yining Zhang, Shengwu Yu, Yanwen Chen, Yuquan Gao, Xiaowei Ma

**Affiliations:** ^1^ Department of Laboratory Medicine, Ningbo Hangzhou Bay Hospital, Ningbo Branch of Renji Hospital, Shanghai Jiao Tong University School of Medicine, Ningbo, Zhejiang, China, shsmu.edu.cn; ^2^ Department of Orthopedics, Ningbo Hangzhou Bay Hospital, Ningbo Branch of Renji Hospital, Shanghai Jiao Tong University School of Medicine, Ningbo, Zhejiang, China, shsmu.edu.cn; ^3^ Central Laboratory, Ningbo Hangzhou Bay Hospital, Ningbo Branch of Renji Hospital, Shanghai Jiao Tong University School of Medicine, Ningbo, Zhejiang, China, shsmu.edu.cn; ^4^ Department of Laboratory Medicine, Renji Hospital, Shanghai Jiao Tong University School of Medicine, Pudong, Shanghai, China, shsmu.edu.cn

**Keywords:** adipogenic differentiation, adipose tissue-derived stromal cells, inflammatory microenvironment, obesity, osteogenic differentiation

## Abstract

**Background:**

Adipose‐derived stem cells (ADSCs), possessing multipotent differentiation potential, hold considerable promise for clinical stem cell therapy applications. ADSCs isolated from obese individuals may exhibit differences compared to those derived from individuals of normal weight.

**Methods:**

Adipose tissues were collected from donors with body mass index (BMI) values within the standard range (Group N) and the obese range (Group O), respectively. ADSCs were isolated from these tissues. The proliferative capacity, phenotypic characteristics, and functional properties of the derived ADSCs were comparatively assessed. To mimic the inflammatory microenvironment of adipose tissue in obese individuals in vitro, ADSCs from Group N were prestimulated with inflammatory cytokines and designated as Group Pre, followed by assessment of their phenotypic characteristics and functional properties. Proteomic analysis was performed on the conditioned medium collected from the ADSCs cultures.

**Results:**

ADSCs of Group N exhibited stronger proliferation capacity than Group O. However, Group N demonstrated lower adipogenic and osteogenic differentiation potential compared to Group O, along with reduced expression of stemness‐associated surface markers and genes. Compared to Group N, Group Pre showed no significant change in proliferative capacity; however, it exhibited enhanced adipogenic and osteogenic potential. Both the surface markers and gene expression associated with stemness shifted toward a more stem‐like phenotype. Proteomic analysis of culture supernatants revealed differentially expressed proteins between Group N and Group O related to cellular basal metabolism, which were also enriched in functions associated with inflammatory and immune responses.

**Conclusion:**

Regarding stem cell characteristics, ADSCs isolated from the human lumbar subcutaneous adipose tissue in obese individuals may exhibit enhanced differentiation potential compared to those derived from individuals of standard weight. This phenomenon may be attributed to the infiltration of inflammatory factors within the pathophysiological microenvironment of the former. This finding provides a novel strategy for autologous stem cell‐based therapies.

## 1. Introduction

In recent years, the clinical application and developmental potential of stem cell therapy have garnered increasing attention. The primary types of stem cells utilized in such therapies include bone marrow‐derived mesenchymal stem cells (BMSCs), human umbilical cord‐derived mesenchymal stem cells (HUMSCs), and adipose‐derived stem cells (ADSCs). Among these, ADSCs hold significant promise in the field of regenerative medicine and may help overcome certain limitations associated with BMSCs and HUMSCs. The clinical harvesting of BMSCs is constrained by limited availability, technical challenges, and highly invasive procedures, often causing considerable patient discomfort [1]. HUMSCs, while a valuable resource, also face scarcity and are frequently accompanied by ethical concerns. In contrast, ADSCs offer several advantages, such as abundant tissue availability, ease of extraction, minimal invasiveness, and the absence of ethical complications when using autologous sources [2]. And there are relatively few reports of adverse reactions associated with the clinical application of ADSCs.

ADSCs have gained considerable attention as a promising tool in regenerative medicine and are currently being explored for the treatment of a variety of diseases [3]. This interest stems largely from their capacity for multi‐lineage differentiation. Several studies have demonstrated that ADSCs can differentiate into endothelial lineages, suggesting potential applications in clinical vascular reconstruction and regeneration [4, 5]. ADSCs are also capable of differentiating into cardiomyocytes, offering a potential strategy for treating myocardial injury and heart failure, and showing promise for cardiac disease therapies [6, 7]. Additionally, their differentiation into osteoblasts and chondrocytes contributes to bone regeneration and the improvement of knee joint dysfunction [8, 9]. In addition to their differentiation capacity, emerging evidence indicates that ADSCs exert therapeutic effects through crucial paracrine mechanisms, secreting a variety of bioactive factors such as cytokines and growth factors [10–12]. Rinaldo et al. demonstrated that ADSCs possess immunomodulatory properties through the secretion of anti‐inflammatory factors, thereby facilitating tissue repair processes [13]. Furthermore, ADSCs have been shown to secrete neurotrophic factors that promote neural regeneration, highlighting their potential applicability in the treatment of neurodegenerative diseases [14–16]. These properties, among others, contribute to their broad therapeutic effects across various disease models, underscoring the considerable potential of ADSCs in multiple medical fields.

The influence of ADSC source on their clinical applicability has been extensively studied. The use of ex vivo‐expanded ADSCs obtained from healthy donors for clinical treatment constitutes allogeneic stem cell therapy. In contrast, the isolation and expansion of ADSCs from the patient themselves, followed by autologous transplantation, can reduce the risk of immune rejection reactions and increase the success rate of clinical treatment, thereby making it a more desirable option in many cases [17]. Obesity represents a growing global health concern and is associated with a range of chronic clinical diseases [18]. ADSCs hold promising potential for the treatment of obesity‐related conditions [19]. Moreover, the abundant adipose tissue availability in obese individuals makes them ideal candidates for autologous ADSC transplantation therapies. Clinical studies have increasingly focused on functional differences in ADSCs derived from individuals with varying degrees of obesity. Most reports indicate that ADSCs from healthy donors exhibit superior performance in multiple stem cell functions, while pathological factors associated with obesity may impair ADSC functionality [20]. However, conflicting findings have also been documented. For instance, one study revealed that ADSCs from obese individuals express significantly higher levels of adipogenesis‐related lncRNAs compared to those from nonobese donors [21]. Furthermore, another study highlighted functional heterogeneity in mesenchymal stem cells derived from different adipose depots between obese and lean individuals [22]. In previous studies, ADSCs were predominantly isolated from abdominal subcutaneous adipose tissue obtained through procedures such as liposuction [23], bariatric surgery [24, 25], or laparoscopic surgery [26], as well as from visceral adipose tissue collected during elective abdominal surgeries [27]. In contrast, the present study utilizes subcutaneous adipose tissue acquired from elective lumbar spine surgery. This source represents a distinct type of adipose tissue that is metabolically less active, exhibits a higher degree of fibrosis, and is anatomically adjacent to skeletal muscle compared to abdominal subcutaneous or visceral fat [28]. We conducted a functional characterization of ADSCs derived from both healthy and obese individuals from this lumbar subcutaneous depot, aiming to identify potential differences and elucidate the underlying molecular mechanisms.

## 2. Materials and Methods

### 2.1. Tissue Samples

Six cases of cryopreserved lumbar adipose tissue specimens, collected and archived by the Biobank of Ningbo Hangzhou Bay Hospital (Ningbo Branch of Renji Hospital, Shanghai Jiao Tong University School of Medicine) between January 2023 and December 2023, were randomly selected for this study. Adipose tissue specimens were classified into two groups according to the World Health Organization (WHO) body mass index (BMI) classification criteria for adults. Three samples derived from individuals with a BMI within the normal range (18.5 kg/m^2^ ≤ BMI ≤ 24.9 kg/m^2^) were designated as the normal group (Group N). Another three samples from individuals classified as obese (BMI ≥ 30.0 kg/m^2^) were designated as the obesity group (Group O). All adipose tissue samples were obtained from adult patients (40 < age of years < 50) undergoing their first open posterior lumbar surgery for lumbar disc herniation. The donors were otherwise healthy with no underlying chronic conditions such as hypertension or diabetes, no history of cancer, and no prior surgical interventions, aside from the diagnosis of lumbar disc herniation. Subcutaneous adipose tissue (~1 cm^3^) was aseptically collected by the same surgeon following skin incision and placed into a sterile EP tube. The samples were then cryopreserved by the Biobank of Ningbo Hangzhou Bay Hospital according to standard collection and preservation protocols.

### 2.2. ADSCs Isolation and Culture

Adipose tissues were incubated in a 5x (2 mg/mL) collagenase type I solution (MilliporeSigma, USA) and minced into a homogenate using sterile scissors. The homogenate was subjected to digestion in a 37°C water bath for 40 min, followed by centrifugation to collect the pellet. After washing the pellet twice, it was resuspended in culture medium and incubated at 37°C in a cell culture incubator with 5% CO_2_. When the extracted cells reached 80% confluency, they were detached using trypsin (Thermo Fisher Scientific, USA) and subsequently subcultured. The resulting cells, designated as passage 1 (P1), were identified as ADSCs. One set of Passage 1 (P1) cells from Group N was continuously stimulated by supplementing the basal culture medium with 50 ng/mL IFN‐γ (R&D Systems, USA) and 10 ng/mL TNF‐α (R&D Systems, USA) and was designated as the Normol‐prestimulation group (Group N‐pre).

### 2.3. ADSCs Proliferation

The proliferative capacity of ADSCs was assessed using a CCK‐8 assay kit (Dojindo, Japan). ADSCs were trypsinized, counted, and seeded at a density of 500 cells per well in a 96‐well plate. After incubation at 37°C in a cell culture incubator with 5% CO_2_ for 12, 24, 48, or 72 h, 10 µL of CCK‐8 reagent was added to each well. The plates were further incubated for 2 h, and the optical density (OD) at 450 nm was measured using a microplate reader (BioTek, USA).

### 2.4. ADSCs Adipogenic and Osteogenic Differentiation

ADSCs were seeded into 6‐well plates and allowed to reach ~75% confluency. The cells were then cultured in induction differentiation medium for 21 days at 37°C in a cell culture incubator with 5% CO_2_, followed by staining to assess the extent of differentiation. Adipogenic induction medium: 10 μM insulin (Beyotime, China), 0.5 μM IBMX (3‐Isobutyl‐1‐methylxanthine), 200 μM indomethacin, and 1 μM dexamethasone. Osteogenic induction medium: 100 nM dexamethasone, 10 mM beta‐glycerol phosphate disodium salt pentahydrate (Solarbio, China), and 50 μM L‐ascorbic acid 2‐phosphate (MCE, China). IBMX, indomethacin, and dexamethasone were purchased from Shanghai Yuanye Bio‐Technology Co., Ltd. (Shanghai, China). The extent of adipogenic differentiation in ADSCs was assessed by staining using Oil Red O Stain Kit (Solarbio, China). The staining procedure for assessing osteogenic differentiation of ADSCs was performed as follows: Cells were fixed with 4% paraformaldehyde (Solarbio, China) at room temperature for 30 min, followed by washing with ddH_2_O. 2% Alizarin Red S staining solution (Beyotime, China) was added, and the cells were incubated for 1 h at room temperature under light‐protected conditions. After removal of the staining solution, the cells were thoroughly washed until no unbound dye could be detected. Images were acquired under a light microscope for analysis.

### 2.5. Flow Cytometry

The expression of stem cell‐associated surface markers on ADSCs was analyzed. For staining, 200 μL of cell suspension was incubated with 5 μL of antibody for 45 min at 4°C under light‐protected conditions. The following antihuman antibodies were used: CD45‐FITC (Biolegend, USA), CD34‐FITC (Biolegend, USA), CD44‐FITC (Biolegend, USA), and CD105‐FITC (Biolegend, USA). Stained cells were assayed and quantified using the Flow Cytometer (Agilent, USA).

### 2.6. Real‐Time PCR Analysis

The total RNA of ADSCs was extracted using RNAeasy Animal RNA Isolation Kit with Spin Column (Beyotime, China) and reverse‐transcribed to cDNA using PrimeScript RT Master Mix (TAKARA, USA). Gene expression levels were evaluated using TB Green Premix Ex Taq (TAKARA, USA) and Applied Biosystems 7500 Real‐Time PCR System (Thermo Fisher Scientific, USA). GAPDH was selected as the internal reference gene. The following primers were used: C/EBPα‐F: 5^′^‐AAGGTGCTGGAGCTGACCAG‐3^′^; C/EBPα‐R: 5^′^‐AATCTCCTAGTCCTGGCTCG‐3^′^; ALPL‐F: 5^′^‐CGAGATACAAGCACTCCCACTTC‐3^′^; ALPL‐R: 5^′^‐CTGTTCAGCTCGTACTGCATGTC‐3^′^; RUNX2‐F: 5^′^‐GACTGTGGTTACCGTCATGGC‐3^′^; RUNX2‐R: 5^′^‐ACTTGGTTTTTCATAACAGCGGA‐3^′^; BMP2‐F: 5^′^‐CCCCCTATATGCTCGACCTG‐3^′^; BMP2‐R: 5^′^‐CCTCGATGGCTTCTTCGTGA‐3^′^; OCT4‐F: 5^′^‐AGCAACTCCGATGGGGCCTCC‐3^′^; OCT4‐R: 5^′^‐GCCCCACATCGGCCTGTG‐3^′^; SOX2‐F: 5^′^‐GCGGAGTGGAAACTTTTGTCC‐3^′^; SOX2‐R: 5^′^‐GGGAAGCGTGTACTTATCCTTCT‐3^′^; Dnmt3b‐F: 5^′^‐CGTTAATGGGAACTTCAGTGACC‐3^′^; Dnmt3b‐R: 5^′^‐CTGCGTGTAATTCAGAAGGCT‐3^′^; GAPDH‐F: 5^′^‐TGACCTCAACTACATGGTCTACA‐3^′^; GAPDH‐R: 5^′^‐CTTCCCATTCTCGGCCTTG‐3^′^. Relative gene expression values were calculated by the ΔΔCt method and normalized.

### 2.7. Proteomics

Cell culture supernatant samples were processed for 4D data independent acquisition (DIA) by Jingjie PTM BioLab (HangZhou) Co., Inc. Protein extraction was performed on the cell culture supernatant samples, and protein was used for trypsin digestion to prepare peptide solution. The peptide segments were dissolved in the mobile phase A of the liquid chromatography system and separated on a EASY‐nLC 1200 UPLC system (ThermoFisher Scientific, USA). The separated peptides were analyzed in Orbitrap Exploris 480 with a nano‐electrospray ion source.

### 2.8. Statistical Analysis

The graphs were prepared with GraphPad Prism 8.0.1 (GraphPad Software, CA). The comparisons between two groups were analyzed using an unpaired Student’s *t*‐tests. And *p*  < 0.05 was considered statistically significant.

### 2.9. Ethics Statement

This study was approved by the Ethics Committee of Ningbo Hangzhou Bay Hospital Ethics Committee (Approval Number KY2025‐01). The human tissue samples used in this study were obtained from the the Biobank of Ningbo Hangzhou Bay Hospital (Approval Nnumber LY2023‐08), which had obtained written informed consent from all participants for the collection and storage of samples for research purposes. Because this study involved the use of de‐identified, preexisting biospecimens, the requirement for additional informed consent was waived by the ethics committee. The present study was conducted in accordance with the Declaration of Helsinki.

## 3. Results

### 3.1. ADSCs Morphology and Proliferation

**Figure 1:**
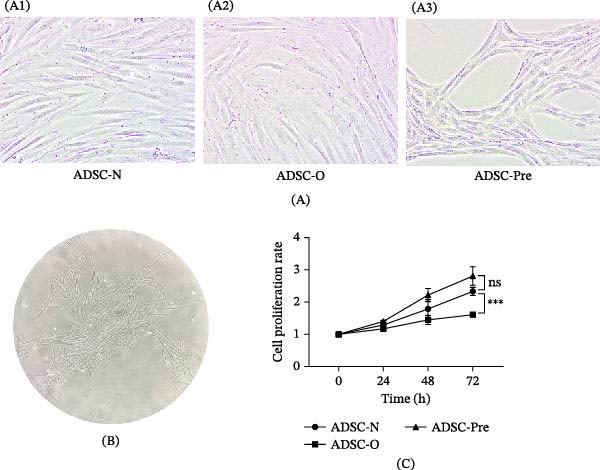
Representative micrograph of cellular morphology and cell proliferation rate of ADSCs. ADSC‐N (A1), ADSC‐O (A2), and ADSC‐Pre (A3) were observed at 200× magnification. ADSC‐Pre (B) were observed at 40× magnification. (C) Cell proliferation rate was assessed at 24, 48, and 72 h. Significant differences were observed between the ADSC‐O group and both the ADSC‐N and ADSC‐Pre groups. Data represent mean±SEM from three independent experiments.  ^∗∗∗^ *p* < 0.001.

When ADSCs were cultured to P1, their morphology was examined under a light microscope. As shown in Figure 1A, no remarkable morphological differences were observed between Group N and Group O. However, Group Pre, stimulated with IFN‐γ and TNF‐α, exhibited a more elongated and spindle‐shaped morphology, appearing “dendritic‐like” under microscopic examination (Figure 1B). We further evaluated the proliferative capacity of ADSCs using the CCK‐8 assay (Figure 1C). The proliferation ability of Group N was significantly stronger than that of Group O (*p*  < 0.05). In contrast, the proliferative capacity of Group Pre was comparable to that of Group N (*p*  > 0.05).

### 3.2. ADSCs Differentiation Ability (Adipogenic and Osteogenic)

**Figure 2:**
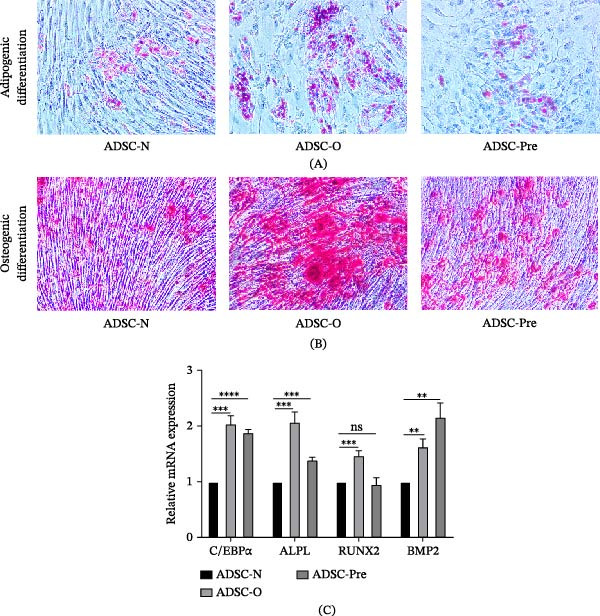
Adipogenic and Osteogenic differentiation ability of ADSCs. (A) Adipogenic differentiation of ADSCs were assessed by Oil Red O and observed under a microscope at 200× magnification. (B) Osteogenic differentiation of ADSCs were assessed by Alizarin Red S staining and observed under a microscope at 200× magnification. (C) The gene expression levels of C/EBPα, ALPL, RUNX2, and BMP2 from ADSCs were analyzed by RT‐PCR. Data represent mean ± SEM from three independent experiments. ^ns^ *p*  > 0.05;  ^∗∗^ *p*  < 0.01;  ^∗∗∗^ *p*  < 0.001;  ^∗∗∗∗^ *p*  < 0.0001.

Following adipogenic and osteogenic induction of ADSCs, differentiation was assessed via histological staining and microscopic examination [29] (Figure 2A,B). In both adipogenic and osteogenic differentiation assays, Group O exhibited a higher degree of differentiation compared to Group N. The differentiation level in Group Pre was intermediate between that of Group N and Group O. The expression of genes associated with adipogenic and osteogenic differentiation was analyzed. As shown in Figure 2C, both Group O and Group Pre exhibited stronger expression than Group N. However, no significant difference in RUNX2 expression was observed between Group N and Group Pre. In contrast, BMP2 expression was higher in the Group Pre compared with Group O, and both groups showed elevated expression relative to Group N.

### 3.3. Stemness Surface Marker Expression in ADSCs

**Figure 3:**
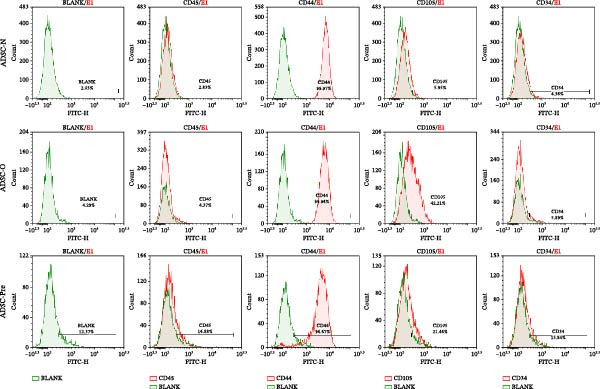
Stemness surface marker expression of ADSCs. ADSCs were adjusted to a density of 1 × 10^6^ cells/mL, incubated with antibodies for staining, and analyzed by flow cytometry to determine positive expression rates. Independent experiments were performed in triplicate.

The expression of stemness‐associated surface markers (CD45, CD44, CD105, and CD34) on ADSCs was examined. As shown in Figure 3, the surface marker profile of ADSCs was characterized by negative expression of CD45 and positive expression of CD44. The percentage of CD105‐positive cells in Group O was significantly higher than that in Group N (*p*  < 0.05), while the expression level of CD105 in Group Pre was intermediate between Group N and Group O. However, the expression level of CD105 was generally low across all three groups, which may be attributed to the fact that the ADSCs were isolated from cryopreserved adipose tissue stored in liquid nitrogen and that the cells used in the experiment were at a relatively high passage number (P5). CD34 was not expressed in any of the three groups.

### 3.4. Expression of Stemness Genes in ADSCs

**Figure 4:**
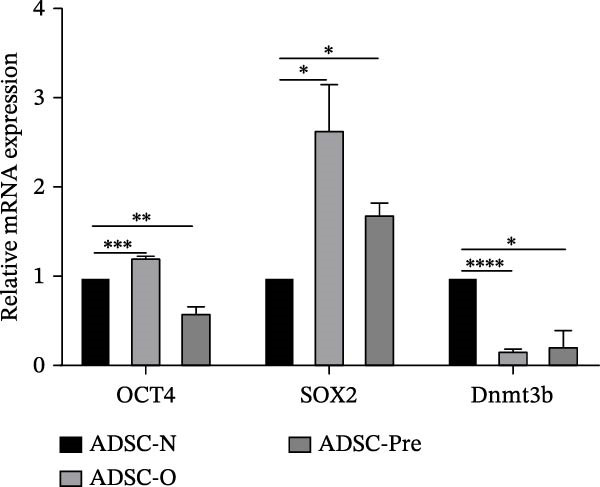
Stemness genes expression of ADSCs. The expression levels of OCT4, SOX2, and Dnmt3b from ADSCs were analyzed by RT‐PCR. Data represent mean ± SEM from three independent experiments.  ^∗^ *p*  < 0.05;  ^∗∗^ *p*  < 0.01;  ^∗∗∗^ *p*  < 0.001;  ^∗∗∗∗^ *p*  < 0.0001.

We selected stem cell‐related genes (OCT4, SOX2, and Dnmt3b) and examined their expression levels in ADSCs. OCT4 and SOX2 are associated with pluripotency and self‐renewal of stem cells. Dnmt3b, involved in methylation, is one of the key genes that promote differentiation and development of stem cells. As shown in Figure 4, the expression levels of OCT4 and SOX2 were higher in Group O compared to Group N, with statistical significance (*p*  < 0.05). In Group Pre, SOX2 expression was also higher than in Group N, while OCT4 expression was lower than that in Group N. Regarding Dnmt3b expression, both Group O and Group Pre showed significantly lower levels compared to Group N (*p*  < 0.05).

### 3.5. Proteomic Analysis of ADSC Culture Supernatant

**Figure 5:**
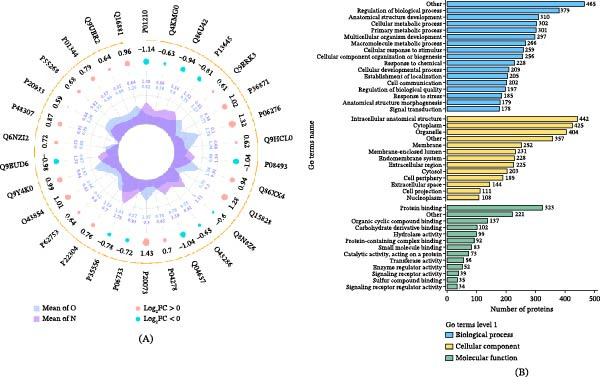
Relative expression levels of the top 30 differentially expressed proteins of ADSCs. Proteomic analysis of culture supernatants from Group N and Group O of ADSCs. (A) Top 30 differentially expressed proteins and their expression profiles and (B) GO secondary classification of the differentially expressed proteins.

**Figure 6:**
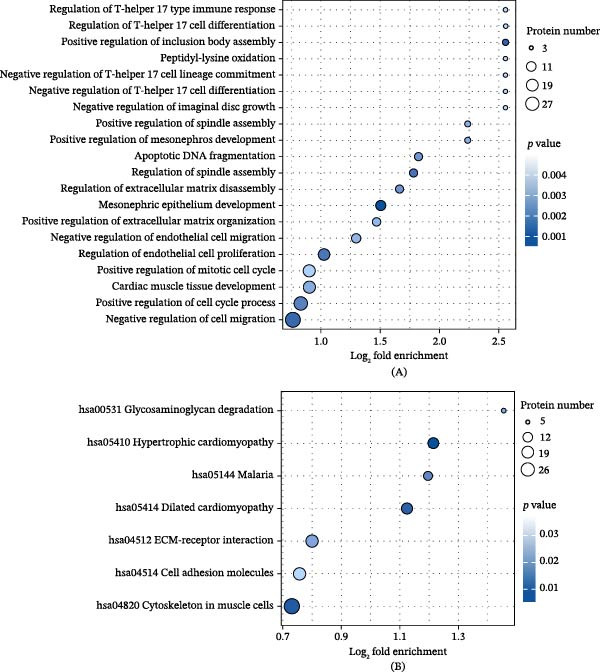
GO enrichment analysis and KEGG pathway analysis of the top 30 differentially expressed proteins of ADSCs. Differentially expressed proteins between Group N and Group O of ADSCs were subjected to (A) GO classification and (B) KEGG pathway enrichment analysis, showing the top 20 most significantly enriched terms.

Proteomic analysis of culture supernatants was derived from Group N and Group O. As shown in Figure 5A, the relative expression levels of the top 30 differentially expressed proteins between the two groups are presented. GO enrichment analysis of the differentially expressed proteins was performed. The results (Figure 5B) indicated that in the biological process category, these proteins were significantly enriched in regulation of primary cellular metabolic process and multicellular organism development. In the molecular function category, the differentially expressed proteins were predominantly enriched in protein binding and organic cyclic compound binding. Regarding cellular component, these proteins were primarily localized to intracellular anatomical structures, the cytoplasm, and organelles. Based on these findings, we further performed enrichment analysis on the differentially expressed proteins in order to determine whether they exhibited significant enrichment trends in specific functional categories. As shown in Figure 6A, GO enrichment analysis revealed that the differentially expressed proteins were significantly associated with regulation of T‐helper 17 cell differentiation and immune response, as well as positive regulation of inclusion body assembly. In the KEGG pathway analysis (Figure 6B), these proteins were strongly associated with hypertrophic cardiomyopathy and dilated cardiomyopathy and were involved in glycosaminoglycan degradation, ECM–receptor interaction, and cell adhesion processes.

## 4. Discussion

In the present study, no visually distinguishable differences in cellular morphology were observed between ADSCs derived from normal‐BMI and obese‐BMI donors. In terms of proliferative capacity, ADSCs from the normal group exhibited stronger growth potential compared to those from the obese group. This observation aligns with the majority of current clinical studies [24], which suggest that stem cells obtained from healthy individuals generally exhibit a more robust and “healthier” phenotype. However, for clinical application, the most valuable aspect of ADSCs is the multidirectional differentiation potential, and we conducted tests on the differentiation ability of ADSCs. Through in vitro induction and culture, we notably observed that ADSCs from obese donors exhibited significantly enhanced adipogenic and osteogenic differentiation capabilities compared to those from normal individuals. This finding appears contrary to the majority of published studies on differentiation potential in abdominal ADSCs [27]. The assessment of adipogenic and osteogenic gene expression (e.g., C/EBPα, ALPL, RUNX2, and BMP2 [30, 31]) yielded results consistent with the above findings, indicating that ADSCs from obese donors possess a stronger capacity to regulate differentiation at the transcriptional level compared to ADSCs from normal individuals. To further investigate this phenomenon, we proceeded to examine stemness‐related characteristics at the molecular level. It is generally accepted that ADSCs positively express markers such as CD44, CD105, CD39, and CD90, while negatively expressing CD45 and CD106 [32, 33]. In the present study, normal‐derived ADSCs showed negative expression of CD105, a stemness‐associated marker, whereas obese‐derived ADSCs exhibited partial positive expression of CD105, suggesting a potential correlation between CD105 expression and the differentiation capacity of ADSCs. According to the surface marker expression criteria for ASCs proposed by the International Society for Cellular Therapy (ISCT) in 2013, the expression levels of CD34 and CD106 are 2% to 30% [32]. Although it has been reported that CD34 positivity may help maintain stem cells in a quiescent state and enhance their regenerative potential [34], our findings indicate that CD34 was not expressed in ADSCs, regardless of their source (normal or obese). ADSCs typically express genes associated with stemness, such as OCT4 and SOX2, which are known to help maintain the undifferentiated state of stem cells. These genes can form molecular complexes that support self‐renewal capacity and multidirectional differentiation potential, serving as key regulators of pluripotency. Notably, ADSCs derived from obese donors exhibited significantly higher expression levels of both OCT4 and SOX2 compared to those from normal individuals, suggesting that, at the genetic level, obese‐derived ADSCs may possess an enhanced multidirectional differentiation potential. It has been reported that omental adipose‐derived ASCs from obese individuals exhibit significantly higher expression of genes such as OCT4, Sal4, and SOX15 compared to those from nonobese donors, indicating that this phenomenon is not exclusive to ADSCs derived from lumbar adipose tissue [35]. The expression of the Dnmt3b gene is associated with de novo DNA methylation and contributes to maintaining stem cells in an undifferentiated state [36]. Studies have shown that both obesity and aging can lead to global DNA hypomethylation [37]. In pluripotent stem cells, hypoxic conditions have been reported to downregulate Dnmt3b expression and promote differentiation [38]. Several epigenetic drugs can inhibit the enzymatic activity of DNMTs, thereby affecting the osteogenic differentiation capacity of ADSCs [39]. In the present study, significantly reduced expression of Dnmt3b was observed in obese‐derived ADSCs, suggesting an enhanced tendency toward differentiation compared to normal‐derived ADSCs, which is consistent with the observed adipogenic and osteogenic phenotypic outcomes.

Obesity induces a systemic inflammatory response, leading to a chronic inflammatory microenvironment within adipose tissue [40]. However, the complexity of the adipose tissue microenvironment in obese individuals cannot be fully recapitulated in vitro. To approximate this condition, we established a simplified pre‐stimulation culture model by supplementing the medium with inflammatory cytokines IFN‐γ and TNF‐α. Normal‐derived ADSCs were cultured under this cytokine‐stimulated condition to investigate how such an inflammatory environment influences their stem cell properties. We observed that pre‐stimulation did not significantly affect the proliferative capacity of ADSCs. However, in terms of the adipogenic and osteogenic differentiation capabilities, the pre‐stimulated ADSCs exhibited a phenotype closer to that of obese‐derived ADSCs, demonstrating stronger differentiation potential compared to normal‐derived ADSCs. This finding is further supported by the work of Zhu et al. [41], who reported that ADSCs from lean pigs exhibited enhanced adipogenic differentiation capacity following TNF‐α treatment. Similarly, although the expression of stemness surface markers in pre‐stimulated ADSCs was not as elevated as in the obese group, it was still significantly higher than that in the normal group. At the gene expression level, pre‐stimulated ADSCs showed patterns similar to obese‐derived ADSCs in the expression of SOX2 and Dnmt3b. However, the expression level of OCT4 was lower than that in normal‐derived ADSCs. The mechanism by which inflammatory factors in the culture environment affect ADSCs is extremely complex. Although the downregulation of OCT4 appears inconsistent with the enhanced differentiation capacity observed in pre‐stimulated ADSCs, gene expression is likely modulated by signaling pathways or other molecular mechanisms. Further studies are warranted to elucidate the specific mechanisms by which inflammatory cytokines affect the expression of stemness‐related genes.

In summary, our results indicate that ADSCs derived from the lumbar subcutaneous adipose tissue of obese individuals exhibit enhanced multidirectional differentiation potential. This characteristic may be associated with the inflammatory factors present in their microenvironment, although the specific biomolecules and mechanisms involved in this effect remain unclear. To further investigate this, we performed proteomic analysis on the in vitro culture supernatants of normal‐derived and obese‐derived ADSCs. Comparative analysis of proteins and peptides in the culture supernatants through multiple databases revealed numerous differentially expressed proteins involved in cellular basal metabolism. KEGG pathway analysis further indicated that these proteins were primarily associated with biological processes related to T‐helper 17 (Th17) cells. Notably, previous studies have reported that ADSCs from obese donors promote the activation of Th17 cells, which in turn suppresses adipogenesis and impairs insulin response [42]. These findings suggest that the functional differences between normal‐derived and obese‐derived ADSCs are closely linked to immune modulation and inflammatory responses within their microenvironment. Differentially expressed proteins were predominantly enriched in pathways associated with cardiac diseases. Previous studies have suggested that certain bioactive factors present in the conditioned medium of ADSCs may share common mechanistic pathways with pharmaceuticals used for treating cardiac and immune‐related disorders [43]. Additionally, it has been reported that ADSCs can exert cardioprotective effects and improve cardiac function following myocardial infarction, which may be attributed to their ability to differentiate into endothelial cells and promote angiogenesis [44, 45].

## 5. Conclusion

The differentiation process of ADSCs may be a continuous process of different stages. The efficient early‐stage differentiation may be a response to the excess energy in the environment, or it could be that adipose tissue differentiation ability is inhibited only when obesity reaches a certain stage. Due to its proximity to the spinal column, lumbar adipose tissue is frequently subjected to passive mechanical stimulation. This characteristic may contribute to the enhanced differentiation capacity of ADSCs derived from obese individuals. There is a study on ASCs derived from the infrapatellar fat pad that shows a similar result to ours, namely, that obese individuals have a more significant differentiation ability than lean individuals [22]. Interestingly, both adipose depots have the characteristics of being proximity to bone and being frequently active, which may contribute to their distinct functional characteristics. This study represents the first investigation into ADSCs derived from the posterior lumbar subcutaneous adipose tissue. It is expected to become an effective treatment method in the autologous infusion of ADSCs for stem cell therapy in obese patients. However, the ADSCs used in this study were derived from frozen tissue, and factors such as tissue size at the time of freezing, freezing method, and freezing duration may all influence cell viability and function, representing a limitation of this research. In recent years, several studies have investigated the viability and function of cells isolated from frozen versus fresh tissues. For instance, Hughes et al. [46] reported that T cell function in mucosal tissues cryopreserved with DMSO was comparable to that in fresh tissues. Dupont et al. [47] found no significant difference in the degree of DNA methylation between frozen and fresh cardiac tissue. Furthermore, Campos et al. [48] observed that while the viability of frozen follicular tissue was reduced, certain functions, such as steroidogenic capacity, were not significantly different from those of fresh tissue; additionally, the duration of cryopreservation did not significantly affect tissue viability or steroidogenic capacity. Considering the potential effects of cryopreservation, we will conduct further studies using ADSC derived from fresh tissue. Otherwise, it should be noted that the different collection methods, collection sites, and culture conditions of ADSCs in clinical practice can all lead to variations in the treatment efficacy of ADSCs, which could compromise their therapeutic efficacy. Collectively, ADSCs provide a strategic basis for treating autoimmune and chronic inflammatory diseases, and hold considerable promise in the fields of tissue engineering and regenerative medicine [49–51].

## Author Contributions

**Yining Zhang:** conceptualization, writing original draft, funding acquisition. **Shengwu Yu:** validation, investigation, visualization. **Yanwen Chen:** methodology, software. **Yuquan Gao:** data curation, formal analysis. **Xiaowei Ma:** project administration, supervision.

## Funding

This work was supported by grants from Ningbo Health Science and Technology program (Grant 2023Y97) and the Medical and Health Science and Technology project of Zhejiang Province (Grant 2025KY308).

## Conflicts of Interest

The authors declare no conflicts of interest.

## Data Availability

Data from this study are available upon request from the corresponding author.
